# Mitochondrial quality control and transfer communication in neurological disorders and neuroinflammation

**DOI:** 10.3389/fimmu.2025.1542369

**Published:** 2025-04-28

**Authors:** Yinrui Ma, Rui Song, Chenyang Duan

**Affiliations:** Department of Anesthesiology, The Second Affiliated Hospital of Chongqing Medical University, Chongqing, China

**Keywords:** mitochondrial quality control, mitochondrial transfer, neurological disorders, neuroinflammation, mitochondrial

## Abstract

Mitochondria, as the primary energy factories of cells, play a pivotal role in maintaining nervous system function and regulating inflammatory responses. The balance of mitochondrial quality control is critical for neuronal health, and disruptions in this balance are often implicated in the pathogenesis of various neurological disorders. Mitochondrial dysfunction not only exacerbates energy deficits but also triggers neuroinflammation through the release of damage-associated molecular patterns (DAMPs), such as mitochondrial DNA (mtDNA) and reactive oxygen species (ROS). This review examines the mechanisms and recent advancements in mitochondrial quality control in neurological diseases, focusing on processes such as mitochondrial fusion and fission, mitophagy, biogenesis, and protein expression regulation. It further explores the role of mitochondrial dysfunction and subsequent inflammatory cascades in conditions such as ischemic and hemorrhagic stroke, neurodegenerative diseases and brain tumors. Additionally, emerging research highlights the significance of mitochondrial transfer mechanisms, particularly intercellular transfer between neurons and glial cells, as a potential strategy for mitigating inflammation and promoting cellular repair. This review provides insights into the molecular underpinnings of neuroinflammatory pathologies while underscoring the translational potential of targeting mitochondrial quality control for therapeutic development.

## 1 Introduction

Mitochondria, the central powerhouses of cells, are indispensable for energy metabolism, maintaining cellular metabolism through ATP production by oxidative phosphorylation (OXPHOS). In addition to their well-known metabolic role, mitochondria regulate key processes such as apoptosis, calcium homeostasis, immune signaling, and the production of reactive oxygen species (ROS). These functions are critical for maintaining cellular homeostasis, especially in the nervous system, where neurons have high energy demands to sustain synaptic transmission, neuroplasticity, and repair mechanisms (1, 2). Given the significant energy demands of neurons, mitochondrial health and function are of particular importance for the survival and proper functioning of the nervous system.

Mitochondrial quality control (MQC) encompasses a range of tightly regulated processes, including mitochondrial fusion and fission, mitophagy, and mitochondrial biogenesis. These processes ensure the removal of defective mitochondria, prevent mitochondrial dysfunction, and maintain an optimal population of healthy mitochondria within the cell (3). However, when mitochondrial quality control is impaired, dysfunctional mitochondria accumulate, leading to the release of mitochondrial DNA (mtDNA) and ROS. These molecules act as damage-associated molecular patterns (DAMPs), which can activate innate immune responses and contribute to chronic neuroinflammation, a key feature in various neurological disorders (4).

Current understanding of MQC in neurodegenerative and neuroinflammatory diseases has advanced significantly over the past decade. Studies have revealed that mitochondrial dysfunction plays a central role in the pathogenesis of neurological diseases such as Alzheimer’s disease, and Parkinson’s disease (5, 6). In these diseases, mitochondrial fusion-fission dynamics are disrupted, mitophagy is insufficient, and excessive ROS production leads to widespread neuroinflammation and neuronal damage. Furthermore, recent research has highlighted the role of intercellular mitochondrial transfer, whereby mitochondria or their components can be transferred between cells to modulate inflammation and promote repair, offering new insights into potential therapeutic strategies (7). Despite these advancements, many critical questions remain unresolved: What are the precise mechanisms by which impaired mitochondrial quality control contributes to neurodegeneration? How can we harness the therapeutic potential of intercellular mitochondrial transfer to alleviate neuroinflammation and repair neuronal networks? These need to be explored in depth.

This review aims to explore the mechanisms of mitochondrial quality control and their role in the pathogenesis of neurological disorders. Specifically, it will summarize recent studies on how mitochondrial dysfunction contributes to neuroinflammation, highlight the importance of maintaining mitochondrial quality in neurons, and examine the emerging therapeutic potential of targeting mitochondrial quality control and intercellular mitochondrial transfer to modulate inflammation and promote neural repair. It aims to contribute to a deeper understanding of mitochondrial quality control’s critical role in neurological diseases and its potential as a therapeutic target.

## 2 Fundamental mechanisms of mitochondrial quality control

**Figure 1:**
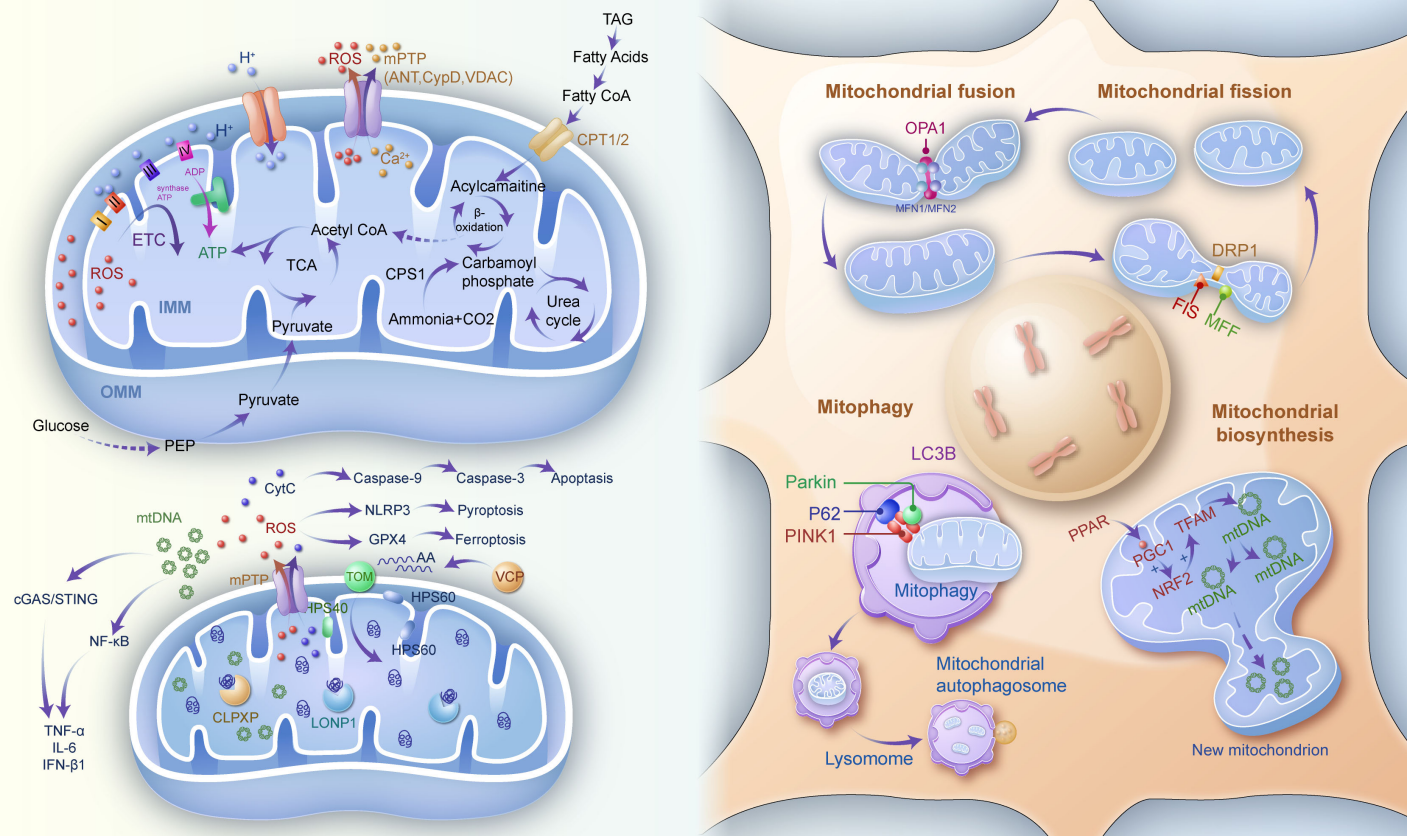
Mitochondrial quality control (e.g., mitochondrial biosynthesis, function and metabolism, mitochondrial fission and fusion as well as mitophagy), and mitochondrial-mediated inflammatory responses. IMM inner mitochondrial membrane, OMM outer mitochondrial membrane, ETC electron transfer chain, ADP adenosine diphosphate, ATP adenosine triphosphate, TCA tricarboxylic acid, ROS reactive oxygen species, mPTP mitochondrial permeability transition pore, ANT adenosine nucleotide translocase, CYPD cyclophilin D, VDAC voltage-dependent anion channel, TAG triacylglycerol, Co A coenzyme A, CPT1/2 carnitine palmitoyl transferase 1/2, CPS1 carbamoyl-phosphate synthase 1, PEP Phosphoenolpyruvate, Cyt C cytochrome C, HSP heat shock protein, NLRP3 NOD-like receptor protein3, GPX4 glutathione peroxidase 4, VCP valosin containing protein, CLPXP caseinolytic protease X and protease P complex, LONP1 lon peptidase 1, mtDNA mitochondrial DNA, TOM translocase of outer mitochondrial membrane, OPA1 optic atrophy protein 1, MFN mitofusin, DRP1 dynamin-related protein 1, FIS mitochondrial fission protein, MFF mitochondrial fission factor, LC3B microtubule associated protein 1 light chain 3 beta, P62 sequestosome 1, PINK1 PTEN induced kinase 1, PPAR peroxisome proliferator activated receptor, PGC progastrcsin, NRF2 nuclear erythroid 2-related factor 2, TFAM transcription factor A, mitochondrial.

Mitochondrial quality control comprises a suite of interconnected mechanisms that maintain mitochondrial function and homeostasis, including fusion and fission dynamics, mitophagy, proteostasis, and biosynthesis. These processes are tightly regulated to ensure that damaged or dysfunctional mitochondria are effectively repaired or eliminated as shown in **Figure 1**.

### 2.1 Mitochondrial dynamics and quality control mechanisms

#### 2.1.1 Dynamic remodeling through fusion-fission balance

Mitochondrial dynamics, regulated by the processes of fusion and fission, are crucial for maintaining mitochondrial integrity and function. Fusion facilitates the mixing of mitochondrial contents, allowing functional recovery and energy redistribution across the mitochondrial network. Mitofusin 1 (MFN1) and Mitofusin 2 (MFN2) mediate fusion at the outer mitochondrial membrane, while optic atrophy 1 (OPA1) controls inner membrane fusion (8). Conversely, fission segregates damaged mitochondria, isolating them for degradation via mitophagy. This process is primarily driven by dynamin-related protein 1 (DRP1), in coordination with adaptor proteins like FIS1, MFF, MID49, and MID51 (9).

An imbalance in fusion and fission dynamics is often implicated in neurological disorders. Excessive fission leads to mitochondrial fragmentation, impaired energy production, and increased susceptibility to apoptosis, while defective fusion limits the mitochondrial network’s ability to compensate for localized damage.

#### 2.1.2 Mitophagy: selective clearance of dysfunctional organelles

Mitophagy, a specialized form of autophagy, is a critical quality control mechanism that removes damaged mitochondria to maintain cellular health. When mitochondrial membrane potential is lost, PTEN-induced kinase 1 (PINK1) accumulates on the outer mitochondrial membrane, recruiting the E3 ubiquitin ligase Parkin. This leads to the ubiquitination of mitochondrial proteins, marking the mitochondria for autophagic degradation (10). Dysregulation of mitophagy has been observed in several neurological disorders, contributing to chronic inflammation and disease progression.

#### 2.1.3 Biosynthesis and proteostasis: replenishment and maintenance

Mitochondrial biosynthesis ensures the replenishment of the mitochondrial population, maintaining both quantity and functionality. This process is regulated by transcriptional coactivators such as PGC-1α, which activates nuclear respiratory factors (NRF1 and NRF2) and mitochondrial transcription factor A (TFAM), facilitating mtDNA replication and transcription (11).

Proteostasis within mitochondria is critical for ensuring proper protein folding, assembly, and degradation. Molecular chaperones like HSP70 and HSP90 assist in protein folding, while degradation systems such as the mitochondrial-associated degradation (MAD) pathway and proteases like Lon and ClPXP remove misfolded or damaged proteins (12). Impaired proteostasis contributes to protein aggregation, a hallmark of neurodegenerative diseases like Alzheimer’s and Parkinson’s disease (2, 13).

### 2.2 Mitochondrial metabolism, homeostasis, and inflammation response

#### 2.2.1 Mitochondrial metabolism and homeostasis

Mitochondria are essential for energy production via oxidative phosphorylation (OXPHOS). OXPHOS is driven by the electron transport chain (ETC) and ATP synthase. The ETC consists of four complexes (I-IV), where Complex I (NADH: ubiquinone oxidoreductase) and Complex II (succinate dehydrogenase) transfer electrons to ubiquinone (CoQ), which then passes electrons to Complex III (cytochrome bc₁ complex) and finally to Complex IV (cytochrome c oxidase) for oxygen reduction to water. Except for Complex II, all other complexes pump protons into the intermembrane space, creating an electrochemical gradient. Protons flow back into the matrix through ATP synthase, driving ATP synthesis from ADP (14). In typical circumstances, the majority of intracellular oxygen is dissociated into water by the transfer of 4 electrons and 4 protons under the action of the ETC complex IV. Approximately 1-2% of the oxygen is reduced to ROS, which are generated to impede the ability to carry electrons, such as CoQ, and thus affect the ability to transfer electrons between complexes I-IV (15). However, the presence of mitochondrial manganese superoxide dismutase (Mn-SOD) in the mitochondria converts the generated ROS to the less reactive H_2_O_2_, which is then converted to water and oxygen by catalase and glutathione peroxidase (GPX), among others (16). However, in pathological states, the oxidative respiratory cascade is impaired, and the excess of ROS produced disrupts the ability of CoQ to carry electrons. This, in turn, disrupts cell signaling and induces oxidative stress (17).

Key regulators of mitochondrial metabolic homeostasis include uncoupling proteins (UCPs) and the mitochondrial permeability transition pore (mPTP) (18). UCPs modulate proton translocation to stabilize membrane potential, while the mPTP, under stress, dissipates membrane potential and releases pro-inflammatory mediators like calcium ions and cytokines (19).

In addition to energy metabolism, mitochondria are central to processes such as fatty acid oxidation, the tricarboxylic acid (TCA) cycle, and lipid synthesis (20). Disruptions in these pathways are often associated with mitochondrial dysfunction and neuroinflammation (21, 22).

#### 2.2.2 Regulation of inflammatory responses and cell death pathways

Mitochondria-derived DAMPs, including mtDNA and cytochrome C, act as potent inducers of inflammation. For instance, mtDNA released into the cytoplasm is recognized by cyclic GMP-AMP synthase (cGAS), which activates the stimulator of interferon genes (STING) pathway, promoting the production of pro-inflammatory cytokines such as IL-6 and TNF (23).Cytochrome C, when released during apoptosis, interacts with apoptotic protease-activating factor 1 (APAF1) to activate Caspase-9, amplifying inflammatory signaling (24).

Excessive ROS production can activate the NLRP3 inflammasome through pathways involving TRAF6 and AMPK, leading to pyroptosis, a form of inflammatory programmed cell death (25). Additionally, mitochondrial dysfunction reduces the expression of mitochondrial transcription factor A (TFAM), exacerbating mtDNA depletion and oxidative respiratory chain disruption. These processes further activate inflammasomes such as AIM2, fueling a vicious cycle of inflammation and mitochondrial damage (26).

ROS accumulation additionally inactivates mitochondrial GPX4, driving lipid peroxidation and ferroptosis (27), a form of oxidative inflammatory cell death (28). Together, these mechanisms underscore the central role of mitochondria in regulating inflammatory responses and their contribution to the pathogenesis of neurological disorders.

Although studies on the interaction of MQC with metabolism and inflammation have revealed its centrality in neurodegenerative diseases, there are still significant limitations in the current research, which constrain the breakthroughs from mechanism resolution to clinical translation. The spatial and temporal synergistic mechanisms of mitochondrial dynamic regulation (fusion/disintegration) and metabolic feedback have not been fully elucidated. For example, how does DRP1-mediated fission respond to changes in energy status (e.g., ATP/AMP ratio) and dynamically regulate autophagy initiation thresholds? Although super-resolution microscopy can resolve mitochondrial network morphology, tools are still lacking for real-time monitoring of the spatiotemporal coupling of mitochondrial autophagy flow, ROS bursts, and inflammatory vesicle activation in living tissues. In addition, the integrated analysis of multi-omics data (e.g. metabolome-epigenome-proteome) is still in the exploratory stage, making it difficult to reveal the synergistic regulatory network of mtDNA variants and epigenomic modifications (e.g. DNA methylation) of the nuclear genome.

## 3 Mitochondrial quality imbalance in neurological disorders and neuroinflammation

The brain is the most energy-demanding organ in the body, consuming over 20% of total energy at rest. This underscores the critical importance of mitochondrial function and integrity for maintaining normal brain operations (29). In central nervous system (CNS) disorders—including hemorrhagic and ischemic stroke, infectious diseases, Alzheimer’s disease (AD), Parkinson’s disease (PD), Huntington’s disease (HD), and brain tumors—dysregulation of mitochondrial dynamics and metabolism leads to mitochondrial stress and structural damage. These pathological changes result in the excessive production of ROS and the release of mtDNA and other mitochondrial DAMPs into the cytoplasm or extracellular environment, triggering neuroinflammation and oxidative stress (2).

**Figure 2:**
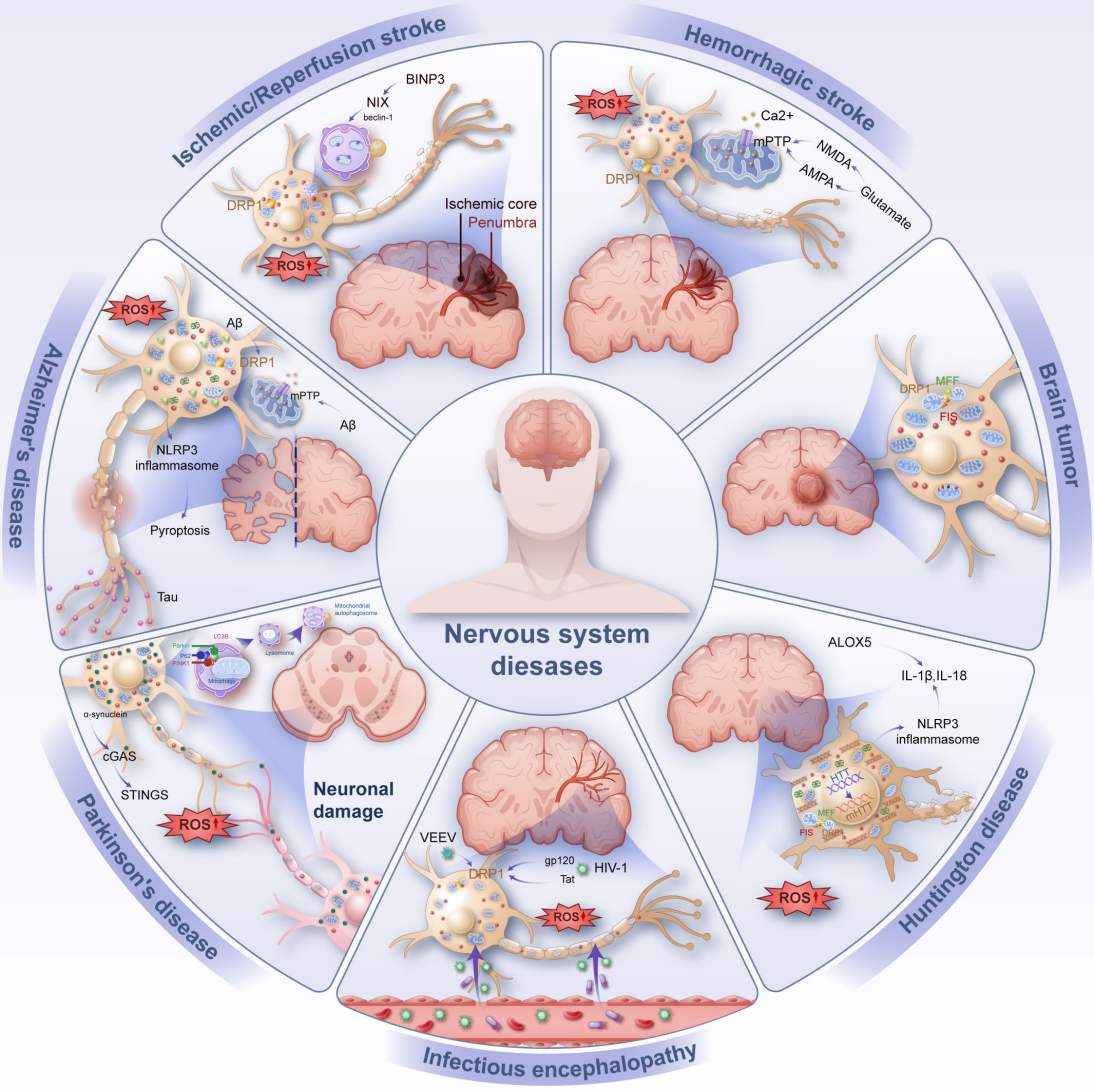
Common inflammation-related diseases of the nervous system (e.g., ischemic/reperfusion stroke, hemorrhage stroke, infectious encephalopathy, Alzheimer’s disease, Parkinson’s disease, Huntington’s disease, brain tumor). Injury manifests as neuronal damage, with intracellular mitochondrial rupture releasing DAMP such as ROS and mtDNA causing oxidative stress and inflammation in neurons. BNIP3 BCL2/adenovirus E1B 19 KDa interacting protein 3, NIX Nip3-like protein X, DRP1 Dynamin-related protein 1, ROS reactive oxygen species, mPTP mitochondrial permeability transition pore, mtDNA mitochondrial DNA, FIS mitochondrial fission protein, MFF mitochondrial fission factor, PINK1 PTEN induced kinase 1, LC3B microtubule associated protein 1 light chain 3 beta, P62 sequestosome 1, Aβ amyloid β-protein, NLRP3 NOD-like receptor protein 3, NMDA N-methyl-D-aspartic acid, AMPA α-amino-3-hydroxy-5-methyl-4-isoxazloe-propioncacid, cGAS Cyclic GMP-AMP synthase, STINGS Stimulator of interferon genes, ALOX5 Recombinant arachidonate-5-lipoxygenase, VEEV Venezuelan equine encephalitis virus, HIV-1 Human immunodeficiency virus 1, HTT Huntingtin.

This process further drives programmed cell death pathways such as ferroptosis and pyroptosis, significantly impacting disease onset and progression. A systematic analysis of mitochondrial quality imbalance in these conditions is essential to deepen our understanding of their pathophysiology and to provide a foundation for mitochondrial-targeted therapies and diagnostic strategies as shown in **Figure 2**.

### 3.1 Mitochondrial quality imbalance in ischemic stroke

Ischemic stroke, caused by cerebral vascular blockage, leads to reduced blood flow, oxygen deprivation, and glucose depletion, resulting in significant neuronal damage. Although reperfusion restores blood supply, it paradoxically generates ROS, which exacerbate brain injury. Mitochondrial quality control plays a pivotal role in mitigating post-stroke neuronal damage. Loss of mitochondrial membrane potential disrupts OXPHOS, reduces ATP production, and causes energy insufficiency, calcium overload, and excessive ROS accumulation, culminating in oxidative stress-induced neuronal injury (30, 31).

Key regulators of mitochondrial dynamics, such as DRP1, are significantly upregulated during oxidative stress, promoting excessive mitochondrial fission and further impairing mitochondrial balance (9). Studies have demonstrated that inhibiting DRP1 reduces infarct size and alleviates neuronal damage in ischemic models (32, 33). Conversely, ischemic events also downregulate mitochondrial fusion proteins such as MFN2, impairing mitochondrial recovery and energy redistribution. Interventions like ginsenoside treatment in ischemia-reperfusion models inhibit MFN2 ubiquitination and degradation, improving mitochondrial fusion and functional expansion (34).

Mitophagy, a critical protective mechanism during ischemia, is activated via factors such as BNIP3 and NIX, which upregulate beclin-1 and initiate the clearance of damaged mitochondria. However, excessive ROS during reperfusion can overactivate mitophagy, exacerbating cell death (31, 35, 36). This dual role of mitophagy necessitates further investigation to optimize therapeutic approaches.

Protective factors such as PPAR, PGC-1α, uncoupling protein 2, and superoxide dismutase 2 (SOD2) also play critical roles in ischemic brain injury. However, oxidative stress suppresses PPAR-s and PPAR-γ activity and hinders PGC-1α activation (37), impairing mitochondrial biogenesis and neuronal recovery (38). Activating PPAR-γ reduces inflammation, while upregulating PGC-1α enhances antioxidant defenses and mitochondrial function (39, 40). In ischemia-reperfusion (I/R) rats, MitoQ treatment restores hippocampal SIRT6 expression, reduces pro-inflammatory mediators like TNF-α and IL-1β, and mitigates mitochondrial oxidative stress (41). Similarly, mitochondrial ferritin (FtMt) has been shown to alleviate iron-driven ferroptosis and neuroinflammation in I/R models (42). Transplantation of healthy mitochondria into I/R mice also suppresses NLRP3-mediated pyroptosis and promotes neurogenesis (43).

### 3.2 Mitochondrial quality imbalance in hemorrhagic stroke

Hemorrhagic stroke, including intracerebral hemorrhage (ICH) and subarachnoid hemorrhage (SAH), results from blood vessel rupture, causing blood infiltration into brain tissue. This mechanical insult compresses surrounding neurons, disrupts mitochondrial dynamics, and induces significant oxidative stress. Mitochondrial quality control mechanisms play an essential role in mitigating the effects of hemorrhagic stroke (44).

Following hemorrhage, mitochondrial swelling, cristae damage, and fragmentation occur, driven by a shift toward excessive fission. Within 24 hours of hemorrhage, DRP1 expression increases significantly, while MFN1, MFN2, and OPA1 levels decrease sharply, with OPA1 showing early reductions within three hours (45–47). DRP1 inhibition and upregulation of MFN1/2 and OPA1 have been shown to ameliorate early brain injury in SAH models (48).

Excessive activation of the PINK1/Parkin mitophagy pathway post-hemorrhage provides partial neuroprotection but may also exacerbate cellular dysfunction, necessitating further exploration of its dual role (49). Hemorrhagic events trigger a surge in glutamate release, activating AMPA and NMDA receptors and causing intracellular calcium overload. This leads to mPTP opening (50), mitochondrial depolarization, and ROS accumulation, further impairing neuronal survival (51, 52).

In ICH, mitochondrial characteristics of ferroptosis include increased iron accumulation and malondialdehyde (MDA) levels, contributing to neuronal death (53). Additionally, mtDNA released as DAMPs activates inflammatory pathways, including the cGAS-STING and NLRP3 inflammasomes, aggravating neuroinflammation (54, 55). Interventions such as oxytocin have shown efficacy in reducing excessive mitochondrial fission, ROS accumulation, and NLRP3-mediated pyroptosis in ICH models (56).

### 3.3 Mitochondrial quality imbalance in CNS infections

Central nervous system infections, caused by viral or bacterial pathogens, elicit intense inflammatory responses that further compromise mitochondrial function. As key regulators of immune responses, mitochondria undergo structural and functional changes in response to infection. Pathogen-associated molecular patterns (PAMPs) and inflammatory mediators disrupt mitochondrial dynamics, leading to ROS accumulation and neuronal damage (31, 57, 58).

For example, rabies virus infection increases mitochondrial complex I and IV activity, enhancing ROS production and oxidative stress (59). Similarly, Venezuelan equine encephalitis virus (VEEV) upregulates DRP1, alters mitochondrial dynamics, and activates mitophagy, ultimately leading to apoptosis in infected astrocytes (57). HIV-1 proteins gp120 and Tat promote mitochondrial fission via DRP1 while impairing mitophagy flux, exacerbating neuronal damage (60–63).

In septic mice, HIF-1α inhibition significantly alleviates mitochondrial damage, reduces hippocampal inflammatory cytokines, and improves neuroinflammation (64). In the sepsis model, NRF2 knockdown led to elevated expression of DRP1 and FIS1, which resulted in overactivation of mitochondrial fission, increased ROS generation, and significant oxidative stress in neurons. Conversely, the upregulation of NRF2 expression led to the reversal of these effects and the upregulation of SLC7A11, thereby preventing the occurrence of iron death, which is caused by iron accumulation in neurons (65). Similarly, targeting mitochondrial pyruvate dehydrogenase reduces M1 microglial activation and alleviates pyroptosis (66), underscoring the interplay between mitochondrial quality control and inflammatory responses in CNS infections (67).

Bacterial infections such as streptococcal meningitis also lead to mitochondrial dysfunction, as lactate accumulation disrupts energy metabolism. Although the exact mechanisms linking mitochondrial quality control and CNS infections remain unclear, further research is critical to elucidate how mitochondrial damage exacerbates inflammation in infectious neurological diseases (68).

### 3.4 Mitochondrial quality imbalance in Alzheimer’s disease

Mitochondrial dysfunction and neuroinflammation are increasingly recognized as early contributors to Alzheimer’s disease (AD) pathogenesis. While amyloid-beta (Aβ) deposition is a hallmark of AD, mitochondrial fragmentation and impaired clearance of damaged mitochondria may act as upstream drivers of neurodegeneration (69, 70).

Reduced expression of mitochondrial fusion proteins (OPA1, MFN1, MFN2) and elevated levels of fission mediators such as DRP1 are common in AD. Excessive mitochondrial fission, triggered by Aβ-induced DRP1 activation, promotes synaptic loss, ROS overproduction (9, 71), and neuronal death (72). Pharmacological inhibition of DRP1 with Mdivi-1 has demonstrated neuroprotective effects by preserving mitochondrial integrity and reducing oxidative stress (73, 74).

Mitophagy, a critical mechanism for removing damaged mitochondria, is severely impaired in AD. PS1 mutations and Aβ accumulation disrupt autophagosome-lysosome fusion, resulting in defective mitophagy and the buildup of inflammatory mediators (75–77). The release of DAMPs, including mtDNA and cytochrome C, further activates neuroinflammation via the NLRP3 inflammasome and other pathways (78).

In addition to mitochondrial dynamics, Aβ plaques directly impair calcium homeostasis by increasing mitochondrial calcium uptake and promote ROS production, which in turn activates mPTP opening, leading to mitochondrial swelling and loss of membrane potential, and ultimately necrotic cell death (79, 80). Targeting proteins such as VDAC1 (81) and NOX4 (82) has been shown to alleviate mitochondrial dysfunction and reduce inflammation in AD models. These findings underscore the central role of mitochondrial quality control in AD progression and suggest potential therapeutic targets.

Furthermore, AD patients exhibit mitochondrial metabolic disturbances, particularly involving tricarboxylic acid (TCA) cycle enzyme activities. For example, the activities of pyruvate dehydrogenase complex (PDHC), isocitrate dehydrogenase (ICDH), and alpha-ketoglutarate dehydrogenase complex (KGDHC) decrease, while succinate dehydrogenase (SDH) and malate dehydrogenase (MDH) activities increase. These enzymatic disruptions closely correlate with the clinical progression of AD and exacerbate mitochondrial dysfunction, contributing to ROS overproduction and neuroinflammation (83).

Laser-capture microdissection reveals significant reductions in mtDNA copy numbers in hippocampal neurons of AD patients, indicating impaired mitochondrial biogenesis signaling. This reduction limits the ability of neurons to maintain mitochondrial homeostasis under pathological conditions (84). Additionally, studies suggest that mutant forms of amyloid precursor protein (APP), such as APP Sweden, suppress the expression of PGC-1α, a key regulator of mitochondrial biogenesis. Restoring PGC-1α levels in AD cell models has been shown to significantly improve mitochondrial and neuronal function, potentially alleviating inflammation and oxidative damage associated with AD progression (85).

### 3.5 Mitochondrial quality imbalance in Parkinson’s disease

Parkinson’s disease (PD) is a progressive neurodegenerative disorder primarily characterized by the loss of dopaminergic neurons in the substantia nigra. Mitochondrial dysfunction, excessive ROS production, and neuroinflammation are central contributors to PD pathogenesis (86, 87). Altered mitochondrial dynamics, bioenergetic failure, and impaired quality control mechanisms collectively exacerbate neuronal damage in PD.

The accumulation of α-synuclein in PD brains disrupts mitochondrial function by impairing electron transport chain (ETC) activity, leading to ATP depletion and increased ROS generation. Elevated levels of oxidized coenzyme Q10 and 8-hydroxy-2’-deoxyguanosine in cerebrospinal fluid further underscore mitochondrial oxidative damage in PD patients (88). Importantly, α-synuclein aggregates activate the cGAS-STING pathway, which amplifies interferon signaling and neuroinflammation. Knockout of STING has been shown to alleviate neuroinflammatory responses and protect against neurodegeneration (89).

Genetic studies have identified mutations in several PD-related genes, including SNCA, PRKN, PINK1, DJ-1, and LRRK2. These mutations disrupt mitochondrial energy metabolism, mitophagy, and ROS homeostasis, highlighting the role of impaired mitochondrial quality control in disease progression (90). The PINK1-Parkin pathway, which mediates mitophagy, is a major regulator of mitochondrial quality in neurons. Loss of mitochondrial membrane potential recruits PINK1 to the outer mitochondrial membrane, where it activates Parkin through phosphorylation (91, 92). Parkin ubiquitinates damaged mitochondria, marking them for autophagic degradation. However, mutations in PINK1 and PRKN impair this pathway, leading to the accumulation of dysfunctional mitochondria and exacerbating neuroinflammation and oxidative stress (93, 94).

Mitochondrial fission and fusion imbalances also play a crucial role in PD. Excessive DRP1-mediated fission fragments the mitochondrial network, promoting neuronal apoptosis and energy deficiency. DRP1 hyperactivation has been observed in PD models, while pharmacological inhibition of DRP1 reduces mitochondrial fragmentation and neuronal loss (92). Mutations in LRRK2, particularly the G2019S variant, further disrupt mitochondrial dynamics by impairing the initiation and completion of mitophagy (95, 96). Increased LRRK2 kinase activity destabilizes mitochondrial homeostasis and promotes neurodegeneration (97).

Mitochondrial DNA (mtDNA) mutations and deletions in dopaminergic neurons are also prominent features of PD. Age-related mtDNA deletions sensitize neurons to oxidative damage, contributing to selective neuronal vulnerability in the substantia nigra (98). PRKN mutations impair mtDNA transcription and replication, leading to progressive mtDNA depletion. This deficiency triggers microglial activation and pro-inflammatory responses, further exacerbating neuronal injury (99).

Therapeutic interventions targeting mitochondrial quality control have shown promise in preclinical models of PD. For example, nicotinamide riboside supplementation improves mitochondrial biogenesis and reduces neuroinflammation by activating PGC-1α signaling (96). In PD mouse models, NBP treatment significantly inhibited mitochondrial PARP1 activation, reduced NLRP3 inflammasome-mediated pyroptosis, and protected dopaminergic neurons by restoring mitochondrial homeostasis (100).

Collectively, these findings underscore the importance of maintaining mitochondrial quality control to mitigate oxidative stress, neuroinflammation, and neuronal loss in PD. Future research should focus on developing targeted therapies that enhance mitochondrial dynamics, mitophagy, and anti-inflammatory responses.

### 3.6 Mitochondrial quality imbalance in Huntington’s disease

Huntington’s disease (HD) is a neurodegenerative disorder caused by CAG repeat expansions in the huntingtin gene (HTT) (101). Mutant HTT impairs mitochondrial transport, increases oxidative stress, and disrupts energy metabolism, leading to neuronal damage (102). Mitochondrial dysfunction in HD is characterized by reduced complex IV activity, ROS accumulation, and decreased mitochondrial content in affected regions like the striatum (103).

Excessive mitochondrial fragmentation in HD is mediated by upregulated DRP1 and FIS1, contributing to ROS production and impaired bioenergetics (104). Mutant HTT also inhibits mitophagy by sequestering autophagic scaffolding proteins, resulting in the accumulation of dysfunctional mitochondria and amplified neuroinflammation (105). This defect in mitochondrial clearance is further exacerbated by progressive mtDNA damage and depletion in HD patients (106–108).

In HD models, iron accumulation and lipid peroxidation drive ferroptosis, a form of oxidative inflammatory cell death. Upregulation of ALOX5 in HD neurons promotes mitochondrial damage and exacerbates oxidative stress (109). Additionally, NLRP3 inflammasome activation and Caspase-1-mediated pyroptosis have been implicated in HD pathology. Suppression of PARP-1 has shown promise in mitigating pyroptotic pathways (110).

Targeting mitochondrial dynamics and reducing oxidative stress hold therapeutic potential in HD. Studies suggest that interventions restoring mitochondrial fusion-fission balance and enhancing mitophagy may alleviate inflammation and neuronal loss in HD (104, 111).

### 3.7 Mitochondrial quality imbalance in intracranial tumors

Mitochondrial dysfunction plays a significant role in the development and progression of intracranial tumors, particularly glioblastoma multiforme (GBM), the most aggressive primary brain malignancy (112). Tumor cells exhibit altered mitochondrial dynamics, ROS overproduction, and metabolic reprogramming to sustain uncontrolled proliferation (113).

Phosphorylation of DRP1 in GBM promotes mitochondrial fragmentation, enhancing tumor invasiveness and resistance to apoptosis (114). Targeting mitochondrial fission with DRP1 inhibitors has shown potential in reducing GBM cell invasion (115). Additionally, disruptions in mitophagy pathways, such as lysosomal-autophagosome fusion inhibition, impair mitochondrial clearance and exacerbate oxidative stress in GBM cells (116).

Mutations in mtDNA further contribute to cancer progression by increasing ROS levels and impairing ETC function. For instance, the A10398G mutation in GBM elevates oxidative stress and promotes tumor cell survival (117, 118). Recent studies have identified mitochondrial-targeted therapies, such as nanoparticles inducing ROS-mediated ferroptosis, as promising strategies for GBM treatment (119).

Exploring the role of mitochondrial quality control and inflammation in intracranial tumors offers new insights into cancer metabolism and potential therapeutic targets for these aggressive malignancies (120).

Despite the critical role of mitochondrial imbalance in neurological disorders, current studies have limitations. Mitochondrial dysfunction involves complex dynamic processes, but their interactions and specific changes in different pathological conditions remain unclear. The heterogeneity of neurological diseases also leads to diverse mitochondrial abnormalities, which existing models fail to fully capture. Moreover, differences between animal models and human diseases limit clinical translation. While therapies targeting mitochondrial function show promise, their long-term effects and safety are still uncertain. Additionally, mitochondrial interactions with other organelles are understudied, hindering a complete understanding of cellular dysfunction. Future research should integrate diverse methods and models to advance both mechanistic insights and clinical applications.

## 4 Mitochondrial transfer and communication in neurons

**Figure 3:**
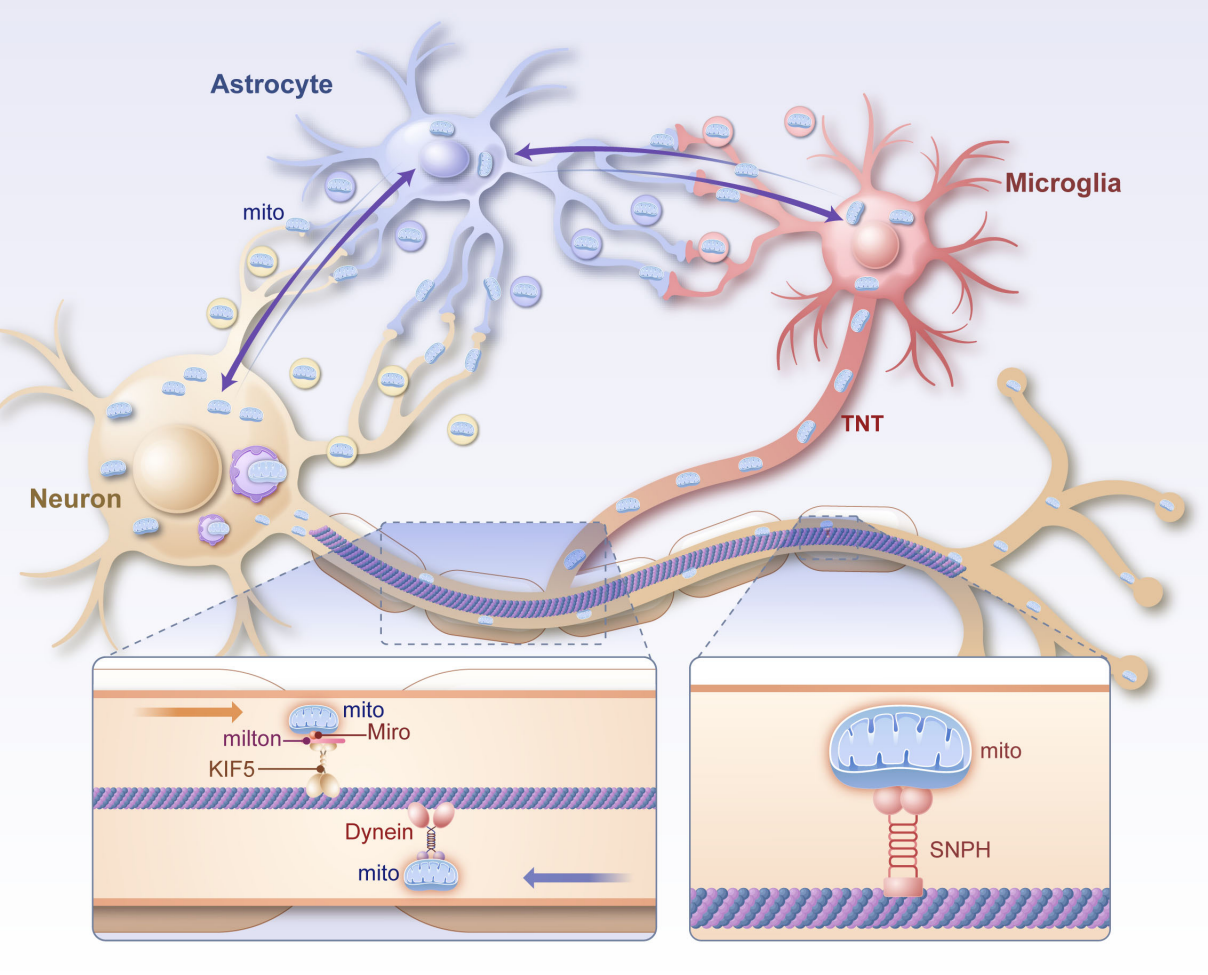
Diagram of the mechanism of mitochondrial transport within neurons and between glial cells. Mitochondria are transported anterogradely from the cytosol to the synapse via the Miro/Milton/KIF5 complex in neuronal axons and retrogradely from the synapse to the cytosol via Dynein. Intercellular mitochondria can be directly transmitted via TNT, vesicles and intercellular contacts. Mito mitochondrion, TNT, tunneling nanotubes, Miro mitochondrial Rho, milton trafficking kinesin protein 1, KIF5 kinesin family member 5, SNPH syntaphilin.

Mitochondrial transfer and communication represent emerging areas of research, focusing on intercellular and intracellular mechanisms that facilitate mitochondrial redistribution and repair in the nervous system. These processes are increasingly recognized as critical for mitigating neuroinflammation, oxidative stress, and energy deficits in neurological disorders as shown in **Figure 3**.

### 4.1 Intracellular mitochondrial transport in neurons

Efficient mitochondrial distribution within neurons is essential for maintaining energy supply and cellular health. Neurons, with their highly polarized structures, rely on robust intracellular transport mechanisms to meet the metabolic demands of axons and synapses. Three primary molecular systems regulate this transport: anterograde transport mediated by TRAK1/TRAK2-MIOR complexes, retrograde transport by dynein motors, and anchoring by syntaphilin (SNPH) in areas of high energy demand, such as synapses and nodes of Ranvier (121, 122).

Approximately 30% of mitochondria in neurons are mobile, supporting dynamic energy redistribution. Following neuronal injury, mitochondria with compromised membrane potential are transported back to the soma for degradation via the PINK1-Parkin pathway (123), while functional mitochondria are delivered to axon terminals to sustain synaptic activity (124). Calcium concentrations modulate mitochondrial anchoring, with elevated levels causing SNPH to release mitochondria from microtubules, enhancing their motility (125). Mutations, such as in the ric-7 gene in C. elegans, demonstrate that improved mitochondrial transport can mitigate neurodegeneration by enhancing axonal energy supply and reducing local oxidative stress (126).

Disruptions in mitochondrial transport are implicated in early neuroinflammatory responses in conditions like traumatic brain injury (TBI). Damaged mitochondria that fail to reach lysosomal degradation sites release cytochrome c and mtDNA, triggering caspase activation and pyroptotic pathways. These inflammatory mediators, including IL-1β and IL-18, exacerbate axonal degeneration (127). Experimental strategies to enhance mitochondrial transport, such as Miro1 overexpression or SNPH deletion (128), have shown promise in restoring mitochondrial distribution, mitigating energy deficits, and alleviating neuroinflammation in axonal injury models (129).

### 4.2 Intercellular mitochondrial transfer between neurons and other cells

Intercellular mitochondrial transfer is a critical mechanism for cellular repair and neuroprotection, particularly in response to oxidative stress and inflammation (130). Mitochondria can be transferred via tunneling nanotubes (TNTs), extracellular vesicles (EVs), endocytosis, or gap junctions, enabling direct exchange of functional organelles between cells (131).

Mesenchymal stem cells (MSCs) play a prominent role in mitochondrial transfer. Under conditions of ischemia or oxidative stress, MSCs transfer healthy mitochondria to injured neurons and astrocytes, restoring ATP production and reducing apoptotic signaling. In neuron-like PC12 cells subjected to hypoxia, mitochondrial transfer from MSCs significantly improves cell survival and function by compensating for mitochondrial dysfunction (132).

Astrocytes also contribute to mitochondrial transfer, supporting neuronal recovery after ischemic injury. Fluorescently labeled astrocyte-derived mitochondria have been observed integrating into neurons, restoring ATP levels and mitigating neuroinflammation. This astrocyte-mediated mitochondrial transfer is crucial for protecting neurons from ischemic damage and reducing oxidative stress (133). However, excessive mitochondrial fission in microglia, mediated by Drp1 and Fis1, can lead to the release of fragmented mitochondria into the extracellular space. These fragments are taken up by astrocytes, driving their transition to the pro-inflammatory A1 phenotype, which exacerbates neuroinflammation in conditions like Alzheimer’s and Parkinson’s diseases (134).

In neurodegenerative diseases, intercellular mitochondrial transfer is vital for managing protein aggregation and inflammation. For instance, mitochondria containing aggregated α-synuclein or tau proteins can transfer from neurons to microglia via TNTs (7). This process activates inflammatory pathways, but reciprocal transfer of healthy mitochondria from microglia to neurons helps restore mitochondrial homeostasis and alleviates neuroinflammation (135). Similarly, TNT-mediated transfer of healthy mitochondria between neurons has been shown to replenish mtDNA and support cellular recovery under oxidative stress conditions (136).

Mitochondrial transfer mechanisms also play a critical role in the tumor microenvironment. In glioblastoma, tumor-associated stromal cells (TASCs) transfer mitochondria to cancer cells via TNTs, promoting glycolysis and conferring resistance to oxidative stress and radiation therapy. These findings highlight the dual role of mitochondrial transfer in both pathological and reparative contexts (137).

Although important progress has been made in the study of mitochondrial transport and communication in the nervous system, many limitations remain. Firstly, mitochondrial transport mechanisms within neurons and between different cells are complex, and their dynamics under different pathological conditions and their regulatory mechanisms are not yet fully understood. Especially in neurodegenerative diseases and brain injury, the specific cellular and molecular mechanisms of impaired mitochondrial transport need to be studied in depth. Although transcellular transfer of mitochondria has shown potential for neuroprotection, e.g., mitochondrial transfer between stem cells and neurons contributes to the repair of damage, the specific role of this process in different cell types and its impact on the inflammatory response remain unclear. Excessive mitochondrial fragmentation and release may also lead to increased inflammation, such as the release of mitochondrial fragments from microglia activating the inflammatory phenotype of astrocytes. Therefore, further exploration of the mechanisms underlying the dual role of mitochondrial translocation in the pathological environment, particularly the balance between disease progression and repair, will help to advance the field further and optimize potential therapeutic strategies.

## 5 Summary and outlook

This review highlights the critical role of MQC in maintaining neuronal health and mitigating the progression of neurological disorders. Key MQC mechanisms, including fission and fusion, mitophagy, biosynthesis, and energy metabolism, are indispensable for preserving mitochondrial homeostasis. Disruptions in these processes often result in mitochondrial quality imbalance, contributing to oxidative stress, energy deficits, and inflammatory responses. Through a systematic analysis, we have examined how MQC dysfunction underlies the pathogenesis of diverse neurological conditions, including ischemic and hemorrhagic stroke, Alzheimer’s disease, Parkinson’s disease, Huntington’s disease, and brain tumors. These findings underscore the interplay between mitochondrial dysfunction and inflammation as a central driver of neurodegeneration.

Emerging research on intercellular mitochondrial transfer provides novel insights into the reparative potential of mitochondrial communication between neurons and glial cells. These processes not only facilitate the removal of damaged mitochondria but also promote neural repair by redistributing functional mitochondria. This expanding area of study offers promising avenues for therapeutic intervention in neurological disorders, particularly in reducing neuroinflammation and oxidative stress.

Mitochondria are more than just energy factories; they are central hubs for cellular signaling, oxidative metabolism, calcium homeostasis, and immune modulation. Their multifaceted roles make them pivotal in the nervous system’s response to injury and disease. Dysfunctional mitochondria release damage-associated molecular patterns (DAMPs) such as mtDNA and cytochrome c, which activate inflammatory pathways like the NLRP3 inflammasome and cGAS-STING, perpetuating neuroinflammation. Understanding the intricate relationship between mitochondrial dysfunction and inflammation will deepen our insights into the mechanisms of neurodegeneration.

Extensive research has highlighted the pivotal role of mitochondria in neuroinflammation, yet the precise mechanisms through which MQC contributes to the pathogenesis of neurological disorders remain inadequately understood. Mitochondria are integral to numerous cellular processes in the nervous system, including energy metabolism, calcium homeostasis, and oxidative stress regulation. However, the specific mitochondrial alterations in various neurological diseases, particularly their interactions with other organelles such as the endoplasmic reticulum and lysosomes, require further investigation. Existing technologies, including oxygen consumption assays and membrane potential measurements, provide valuable insights into mitochondrial metabolism but fall short in enabling dynamic observation of mitochondrial behavior and inter-organelle communication. While emerging imaging techniques such as super-resolution microscopy offer high-resolution structural details, they remain limited in their ability to capture dynamic mitochondrial functions. Moreover, the translational potential of mitochondrial research is hindered by significant differences between animal models and human pathology. To overcome these challenges, future research should prioritize the development of disease-relevant models, advanced methodologies, and a comprehensive understanding of mitochondrial mechanisms in neuroinflammation.

Mitochondrial dysfunction is often an early hallmark of neurological diseases, emphasizing the importance of developing mitochondrial-based biomarkers for early diagnosis, disease progression prediction, and treatment monitoring. These biomarkers hold significant potential for clinical application. Additionally, recent advancements in mitochondrial transfer therapies, which aim to restore cellular energy supply, reduce oxidative stress, and modulate inflammatory responses, show promise in treating conditions such as stroke and traumatic brain injury. Given the unique nature of mitochondria as cellular organelles, there are opportunities to address genetic disorders by correcting mtDNA mutations, allowing for the development of personalized therapeutic strategies based on individual genetic profiles and disease characteristics. Mitochondria-targeted therapies not only offer the potential to alleviate symptoms of neurological diseases but may also fundamentally alter disease progression by restoring mitochondrial function. Enhancing collaboration between basic research and clinical translation, alongside advancing clinical trials, is essential to drive future breakthroughs in the treatment of neurological disorders.

Future research must address several critical challenges and opportunities:

- Deciphering molecular mechanisms: Advances in single-cell technologies, high-resolution imaging, and genetic editing tools are poised to unravel the dynamics of mitochondrial quality control and intercellular communication in neurons. Identifying disease-specific MQC pathways could provide new molecular targets for intervention.
- Biomarker development: Developing mitochondrial-based biomarkers, such as circulating mtDNA fragments or mitochondrial-specific metabolites, holds promise for early diagnosis and disease monitoring in neurological disorders.
- Therapeutic innovation: Targeting mitochondrial dysfunction through pharmacological modulators of MQC pathways, mitochondrial transfer therapies, or gene editing approaches offers a frontier for precision medicine. Restoring mitochondrial function could not only alleviate neuroinflammation but also enhance neuronal survival and function.
- Clinical translation: Preclinical findings must be translated into clinical applications through rigorous trials that evaluate the safety, efficacy, and scalability of mitochondrial-targeted therapies in treating neurological diseases.

In conclusion, mitochondria are at the heart of the intricate interplay between cellular metabolism, inflammation, and neurodegeneration. Continued exploration of MQC mechanisms and intercellular mitochondrial transfer will advance our understanding of neurological disease pathophysiology and open new therapeutic horizons. By integrating basic research with clinical innovation, the future of mitochondrial medicine holds the potential to revolutionize precision diagnostics and individualized treatment strategies for neurological disorders.
